# Genomic prediction of the polled and horned phenotypes in Merino sheep

**DOI:** 10.1186/s12711-018-0398-6

**Published:** 2018-05-22

**Authors:** Naomi Duijvesteijn, Sunduimijid Bolormaa, Hans D. Daetwyler, Julius H. J. van der Werf

**Affiliations:** 1 Cooperative Research Centre for Sheep Industry Innovation, Armidale, NSW 2351 Australia; 2 School of Environmental and Rural Science, University of New England, Armidale, NSW 2351 Australia; 3 Agriculture Victoria, AgriBio, Centre for Agribioscience, Bundoora, VIC 3083 Australia; 4 School of Applied Systems Biology, La Trobe University, Bundoora, VIC 3083 Australia

## Abstract

**Background:**

In horned sheep breeds, breeding for polledness has been of interest for decades. The objective of this study was to improve prediction of the horned and polled phenotypes using horn scores classified as polled, scurs, knobs or horns. Derived phenotypes polled/non-polled (P/NP) and horned/non-horned (H/NH) were used to test four different strategies for prediction in 4001 purebred Merino sheep. These strategies include the use of single ‘single nucleotide polymorphism’ (SNP) genotypes, multiple-SNP haplotypes, genome-wide and chromosome-wide genomic best linear unbiased prediction and information from imputed sequence variants from the region including the *RXFP2* gene. Low-density genotypes of these animals were imputed to the Illumina Ovine high-density (600k) chip and the 1.78-kb insertion polymorphism in *RXFP2* was included in the imputation process to whole-genome sequence. We evaluated the mode of inheritance and validated models by a fivefold cross-validation and across- and between-family prediction.

**Results:**

The most significant SNPs for prediction of P/NP and H/NH were OAR10_29546872.1 and OAR10_29458450, respectively, located on chromosome 10 close to the 1.78-kb insertion at 29.5 Mb. The mode of inheritance included an additive effect and a sex-dependent effect for dominance for P/NP and a sex-dependent additive and dominance effect for H/NH. Models with the highest prediction accuracies for H/NH used either single SNPs or 3-SNP haplotypes and included a polygenic effect estimated based on traditional pedigree relationships. Prediction accuracies for H/NH were 0.323 for females and 0.725 for males. For predicting P/NP, the best models were the same as for H/NH but included a genomic relationship matrix with accuracies of 0.713 for females and 0.620 for males.

**Conclusions:**

Our results show that prediction accuracy is high using a single SNP, but does not reach 1 since the causative mutation is not genotyped. Incomplete penetrance or allelic heterogeneity, which can influence expression of the phenotype, may explain why prediction accuracy did not approach 1 with any of the genetic models tested here. Nevertheless, a breeding program to eradicate horns from Merino sheep can be effective by selecting genotypes *GG* of SNP OAR10_29458450 or *TT* of SNP OAR10_29546872.1 since all sheep with these genotypes will be non-horned.

**Electronic supplementary material:**

The online version of this article (10.1186/s12711-018-0398-6) contains supplementary material, which is available to authorized users.

## Background

Breeding for polled animals is a long-standing issue in horned species such as cattle, sheep and goats [1–3] and analyzing its underlying genetic mechanism is interesting from both a breeding perspective and evolutionary perspective. In natural populations of ruminants, the occurrence of horns serves two major purposes. First, they help an animal to defend itself from predators, and second, horns are important to determine dominance between males and gain access to females. For farmers, the foremost reason to favour polled animals is that horned animals can pose a physical security risk to the farmer, but can also cause injury to other livestock, which results in downgrading of meat due to bruising. Breeding practices can reduce or completely remove the occurrence of horned animals from the population.

In cattle, sheep and goats, several studies have attempted to describe the genetic mechanism that underlies the horned phenotype. In cattle, the most common mutation for the polled phenotype is mapped to the proximal end of bovine chromosome 1, where different genomic variants are described as causative (insertion, duplication, and haplotypes) in different European breeds [4, 5]. Also overexpression of the *FOXL2* and *RXFP2* transcripts in horn buds has been reported in cattle [4], which are genes known to affect or potentially affect horn formation in sheep and goats. In goats, an 11.7-kb deletion located on chromosome 1 was identified as the causative mutation for the polled intersex syndrome (PIS), this deletion also affecting the transcription of two flanking genes, *FOXL2* and a non-coding RNA [6]. A quantitative trait locus (QTL) for polledness in sheep has been mapped to chromosome OAR10 (OAR for *Ovis aries*) [7]. Wiedemar and Drögemüller [8] provided evidence that a 1.78-kb insertion in the 3′-untranslated region of the *RXFP2* gene causes a polled phenotype in sheep. However, Lühken et al. [9] showed that this insertion does not completely explain the polled phenotype in various sheep breeds.

The mode of inheritance of polledness is complex since expression of this phenotype differs between sexes and because no single-locus model with complete penetrance can explain the phenotypic variation both within and across breeds. Moreover, this issue is complicated by the existence of intermediate phenotypes such as knobs and scurs. A knob is a hard bony lump at the horn site which may have a horny cap, generally less than 2.5 cm long, and a scur is a horny growth, irregular in shape and smaller than a true horn [10]. In addition after castration of rams, the development of the horn is stopped or reduced [11], which makes accurate collection of horn scores more difficult.

Breeding strategies have been developed to increase polledness in several sheep breeding programs. Selection on polledness in both males and females (without the presence of genomic data) has been successful and resulted in polled breeds such as the polled Merino and Poll Dorset. With the availability of genomic data, it should be possible to optimize selection for polledness. A clear difference in allele frequency between polled and horned breeds is observed for single nucleotide polymorphisms (SNPs) that are closely linked to the 1.78-kb insertion on OAR10 [12]. Currently, two such SNPs are used by the Australian sheep CRC to predict the phenotype i.e. OAR10_29546872.1 and OAR10_29389966_X.1. These SNPs were chosen from the Illumina Ovine 50k SNP chip, based on their significant association with polled phenotypes in Australian data from the CRC Information Nucleus Flock [13] and the Sheep Genomics Flock [14], respectively. SNPs that are located near the 1.78-kb insertion in the *RXFP2* gene are not present on the Illumina Ovine 50k chip or the OvineHD 600k chip, but four such SNPs have been included in the Illumina 15k Ovine array that was released in 2016.

Currently, prediction accuracy of the horned phenotype is not equal to 1, because the predictive SNP is not in full linkage disequilibrium (LD) with the 1.78-kb insertion, or penetrance is incomplete, or because of epistasis, i.e. interaction between two or more genes. Recently, whole-genome sequence data of 726 European sheep from various breeds have become available. Thus, imputation of the 1.78-kb insertion into the Merino population could provide a better predictor than the current SNPs used for prediction. To further investigate the genetic architecture of the polled and horned phenotypes, we compared models that fit the effects of all SNPs as random effects with the same a priori distribution (genomic best linear unbiased prediction; GBLUP) and the effects of the most predictive SNPs as fixed effects, in order to estimate the variance explained by all SNPs genome wide, conditional on the genotypes of the most predictive SNPs in the *RXFP2* region.

In this study, we evaluated various strategies for predicting horned or polled phenotypes in 4001 purebred Merino sheep, which include the use of single-SNP genotypes, multiple-SNP haplotypes, genome-wide and chromosome-wide GBLUP and information from structural variants in the *RXFP2* region imputed to sequence level.

## Methods

### Population and phenotypic data

Data were extracted from two research datasets known as the Information Nucleus Flock (INF [13]) and the Sheep Genomics Flock (SGF [14]) and consisted of purebred Merino sheep that represented both polled Merino and Merino subtypes. For the analyses, we used 4001 sheep, which were born between 2007 and 2011 and distributed among eight flocks. These sheep originated from 182 sires, which had between one and 51 offspring with dams that had on average 1.5 offspring present in the dataset.

The phenotypes polled, scurs, knobs or horns were recorded. Analysis was performed on two binomial traits that were classified as polled/non-polled (P/NP) and horned/non-horned (H/NH). Table 1 shows the distribution of the polled and horned status between females and wethers (since all male sheep were castrated before horn scoring). Horn scoring was done around three to six months after marking. The frequency of horns was much lower in Merino sheep registered as ‘polled’. Sheep registered as ‘polled’ represented 40% of the data and included 6% horned, 56% polled and 38% sheep with scurs or knobs. Merino sheep not registered as ‘polled’ (60% of the data) included 22% horned, 25% polled and 53% scurs or knobs.

**Table 1:** Number of observed phenotypes for male and female Merinos

	Females	Males (wethers)
Polled	1123	511
Knobs + scurs	1237	561
Horned	88	481

For the derived phenotype horned/non-horned (H/NH), horned animals are contrasted with animals with horns scored as ‘polled’, ‘knobs’, and ‘scurs’ and for the derived phenotype polled/non-polled (P/NP), polled animals are contrasted with animals with horns scored as ‘horned’, ‘knobs’, and ‘scurs’

### Genotypes

Of the 4001 animals, 3708 were genotyped with the OvineSNP50k BeadChip and the remaining 293 were genotyped with the Illumina-Ovine 12k SNP chip. Four hundred and forty-five animals were also genotyped with the Illumina Ovine HD (600k) (Illumina Inc., San Diego, CA, USA). Quality controls for each SNP chip were the same: SNP genotype records were removed if the call rate was less than 90%, if the GC (GenCal) score was less than 0.6, if deviation from Hardy–Weinberg equilibrium (χ^2^ > 600) was strong, and if the minor allele frequency (MAF) was lower than 0.01. In addition, only autosomal SNPs were used for imputation. Finally, 11,377, 48,559 and 510,174 SNPs remained for the 12k, 50k and HD SNP chips, respectively. The 12k genotypes were imputed up to 50k using the Beagle software v3.2 [15] and the 50k genotypes were imputed up to HD using FImpute v2.2 [16]. For both programs, default settings were used. In both imputation steps, all available genotyped animals from INF and the Sheep Genomics Flock were used, to increase imputation accuracy (22,684 animals for the 50k and 2450 for the HD chip). Accuracy of imputation was tested elsewhere [17, 18] and was generally high (on average a correlation of 0.98).

### Statistical analyses

Four different methods were applied to predict the P/NP and H/NH phenotypes.

In method (1), we used a single-SNP genotype to predict the phenotype. To select the best SNP, we ran an association study for all SNPs on OAR10 by fitting each SNP as a covariate. Each single-SNP genotype was either fitted as a single predictor variable (base model), or was fitted jointly with a polygenic effect. This polygenic effect was assumed to be normally distributed with a covariance structure that was proportional to either the numeral relationships expected based on pedigree information or a structure described by a genomic relationship matrix (GRM). To account for the binomial nature of the response variables, we used logistic regression with the statistical package ASReml v4 [19].

In method (2), we used multiple SNPs to predict the phenotype. A haplotype was formed using the most significant SNPs from the single-SNP GWAS using either three, five or ten SNPs. The three haplotypes covered a region of 5, 55 or 125 kb both up- and downstream of the 1.78-kb insertion in the *RXFP2* gene. Haplotypes were formed after phasing the genotyping data using EAGLE v1.0 [20]. Each animal has either no, one or two copies of the available haplotypes. For haplotypes with a frequency lower than 2%, we used one class of residual haplotypes. The number of unique haplotypes found for combinations of three, five or ten SNPs, were equal to 3, 3, and 7, respectively. This limited number of haplotypes for each SNP combination reflects the high LD between the SNPs used. Haplotypes were fitted as random effects, jointly with a polygenic effect (either through a numerator relationship matrix or a GRM).

Method (3) applies a GBLUP analysis using a GRM [21] based on either all SNPs from the HD chip or only the SNPs located on OAR10. In addition, a dominance relationship matrix was added to the model [22] based on these same sets of SNPs using the GCTA package [23]. The statistical software package MTG2 v2.06 [24] was used to predict additive genetic and dominance effects. From the genomic estimated breeding values (GEBV) and dominance genetic values of the animals in the reference set, effects of SNPs were back-solved based on their genotypes, and these SNP solutions were used to estimate the breeding values of the validation animals. Additive and dominance effects were summed across all SNPs to obtain the predicted phenotype.

In method (4), we tried to identify the 1.78-kb insertion described by Wiedemar and Drögemüller [8] based on whole-genome sequence data from 122 Merino sheep sequenced by the SheepCRC project and we imputed this structural variant in the studied population. Whole-genome sequence fastq reads were quality-controlled and aligned with BWA mem as described in Daetwyler et al. [25]. The reference whole-genome sequence in sheep (OAR3.1) was obtained from a polled Texel animal that carries the 1.78-kb insertion. To detect this variant that predicts horned animals, we used two software programs that are suitable for detecting large deletions, i.e. Pindel v0.2.5b9 [26] and Breakdancer v1.1.2 [27]. We ran both programs using the bam files from each of the 122 sequenced Merino sheep. These 122 animals did not have phenotype records and therefore did not overlap with the 4001 Merinos used in this study although they were related to them. The Pindel package uses split-read approaches to identify large deletions. Breakdancer uses discordant paired reads and incorporates the coverage of the reads and the size of the region defined by the discordant pairs into a confidence score. Both programs were run with default settings to call structural variants. Consensus between Pindel and Breakdancer was required to call the 1.78-kb insertion reported by Wiedemar and Drögemüller [8].

Pindel can detect whether an individual carries no, one or two insertions, while Breakdancer only detects the presence of the insertion. Seventy-four animals, which were concordant between Breakdancer and Pindel, were used for imputing the 1.78-kb insertion in the population of 4001 Merino sheep using EAGLE v1.0 for phasing [20] and Minimac3 [28] for imputation. Forty-seven animals were homozygous for the insertion, 10 were heterozygous and 17 were homozygous for absence of the insertion.

### Mode of inheritance

We compared various models to investigate the mode of inheritance (effect of dominance and sex) of the polled and horned phenotypes fitting a single SNP (fitted as random effect):

1 $${\mathbf{y}} = {\mathbf{1}}{\varvec{\upmu}} + {\mathbf{Zu}} + {\varvec{\Lambda}}_{i} \alpha_{i} + {\mathbf{e}},$$

2 $${\mathbf{y}} = {\mathbf{1}}{\varvec{\upmu}} + {\mathbf{Zu}} + {\varvec{\Lambda}}_{i} \alpha_{i} + {\varvec{\Delta}}_{i} \delta_{i} + {\mathbf{e}},$$

3 $${\mathbf{y}} = {\mathbf{1}}{\varvec{\upmu}} + {\mathbf{Zu}} + sex^{*} {\varvec{\Lambda}}_{i} \alpha_{i} + {\mathbf{e}},$$

4 $${\mathbf{y}} = {\mathbf{1}}{\varvec{\upmu}} + {\mathbf{Zu}} + {\varvec{\Lambda}}_{i} \alpha_{i} + sex^{*} {\varvec{\Delta}}_{i} \delta_{i} + {\mathbf{e}},$$

5 $${\mathbf{y}} = {\mathbf{1}}{\varvec{\upmu}} + {\mathbf{Zu}} + sex^{*} {\varvec{\Lambda}}_{i} \alpha_{i} + sex^{*} {\varvec{\Delta}}_{i} \delta_{i} + {\mathbf{e}},$$

where ${\mathbf{y}}$ is a vector of length N (N = 4001) with phenotypic observations for all animals, ${\varvec{\upmu}}$ is the intercept, **Z** is the incidence matrix relating observations to an individuals’ polygenic effects, ${\mathbf{u}}$ is the vector of polygenic breeding values [$N\left( {0,{\mathbf{A}}\upsigma_{\text{A}}^{2} } \right)$, ${\varvec{\Lambda}}_{i}$ is a vector of length N with genotypes for SNP $i$ coded as 0, 1 or 2 representing the three classes of genotypes, $\alpha_{i}$ is the random allele substitution effect at SNP $i$, for which the most significant SNP from the GWAS was used and ${\mathbf{e}}$ is a vector of residuals. In Model 2, a dominance effect was fitted where ${\varvec{\Delta}}_{i}$ is a vector of length N with heterozygous animals at SNP $i$ coded as 1 and homozygous animals as 0, and $\delta_{i}$ being the random dominance effect at SNP $i$. Model 1 was the null model, in which the SNP was fitted as an additive effect. Model 2 included both an additive and dominance effect for the SNP. An interaction term with sex (wether or female) was included in Models 3, 4 and 5 for the additive, or dominance, or both effects, respectively.

Models were compared based on Akaike’s information criterion (AIC [29]). We compared the difference in AIC value (ΔAIC) between models. Model 1 was the null model: fitting an additive effect only. The more the AIC is negative, the better is the model fit. The model, which had the best fit (i.e. the lowest AIC), was used for further analyses. The models described here (1–5) did not allow for incomplete penetrance.

### Validation

A fivefold cross-validation was repeated five times to compare prediction accuracy of the five models. Prediction accuracy was defined as the Pearson correlation between the predicted phenotype in the test set and the binomial traits P/NP or H/NH. The mean of accuracy and the standard deviation across folds are reported.

P/NP and H/NH may not represent fully monogenic traits and we wanted to further investigate the inheritance pattern of these traits. Therefore, we applied another validation strategy that contrasted the use of family information versus no family information in predicting phenotype. The dataset was split by using two different approaches. First, within-family prediction was estimated by using phenotypic data from approximately half of the animals in each half-sib family to predict the phenotypes of the other half of the animals (intra-family comparison). Second, across-family prediction was estimated by using phenotypic data from approximately half of the families to predict the phenotypes of the animals in the other half of the families (inter-family comparison). Sires with more than 13 offspring were selected for family validation. One hundred and thirty-eight sire families and 3671 sheep were used for this validation. The mean of accuracy (Pearson’s correlation) and standard error across a fivefold validation are reported.

## Results

**Fig. 1:**
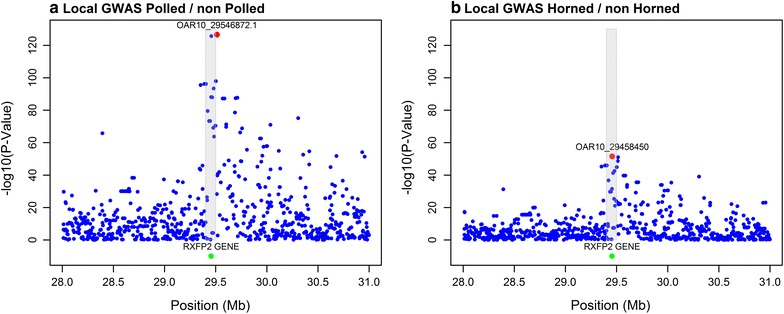
Local genome-wide association plot for the traits **a** polled/non-polled and **b** horned/non-horned of OAR10. The grey rectangle indicates the location of the *RXFP2* gene (29.4–29.5 Mb). The most significant SNP is indicated in red with its name

The local association study for P/NP and H/NH clearly confirmed that the *RXFP2* region is significantly associated with the phenotypes and identified highly significant SNPs at about 29.5 Mb on OAR10 (Fig. 1). The most significant SNP for polled was OAR10_29546872.1 (− log_10_(*p*-value) = 126), which differed from the most significant SNP for horned, i.e. OAR10_29458450 (− log_10_(*p*-value) = 51), although both SNPs are close to each other (0.5 Mb apart, located upstream of the insertion) and in high LD (r^2^ = 0.985). Both SNPs always ranked as number 1 or 2 in terms of significance level, and were used to test the accuracy of prediction for the single SNP analyses. The region around the 1.78-kb insertion also contained other significantly associated SNPs, e.g. in the region between 29 and 30 Mb, the average − log_10_(*p*-value) across all 228 SNPs was equal to 24 for P/NP and 11 for H/NH. Note that, in general, *p*-values less significant for the H/NH phenotype because the frequency of horned phenotypes in the dataset was low, especially in females (Table 1), which resulted in less power to detect SNP effects. Both SNPs were present on the Illumina Ovine HD chip and SNP OAR10_29546872.1 was also present on the OvineSNP50k chip.

In Tables 2 and 3, the proportions of genotypes for the most significant SNP for phenotypes P/NP and H/NH are provided for each sex. All males and females, except one that had genotype 2 for SNP OAR10_29458450, were non-horned (Table 3) although not all are polled (84% of females and 78% of males were polled). The probability of each genotype to carry a particular phenotype was derived simply from the mean of the binary trait for that class of genotypes. The probability of an animal with genotype 0 to be horned is less than 1, and this probability is especially low in females (0.07) compared to males (0.67).

**Table 2:** Proportion of SNP OAR10_29546872.1 alleles per sex for the polled phenotype and probability of animals being polled

Sex	Genotype	Polled (0)	Non-polled (1)	Probability of being polled^a^
Female	0 (*AA*)	174	1058	0.14
	1 (*AT*)	811	353	0.77
	2 (*TT*)	138	25	0.85
Male	0 (*AA*)	29	675	0.04
	1 (*AT*)	385	340	0.53
	2 (*TT*)	97	27	0.78

^a^Based on the proportion of polled animals in each genotype class

**Table 3:** Proportion of SNP OAR10_29458450 alleles per sex for the horned phenotype and probability of animals being horned

Sex	Genotype	Non-horned (0)	Horned (1)	Probability of being horned^a^
Female	0 (*AA*)	1149	81	0.07
	1 (*AG*)	1046	6	0.01
	2 (*GG*)	165	1	0.01
Male	0 (*AA*)	228	472	0.67
	1 (*AG*)	719	9	0.01
	2 (*GG*)	125	0	0.00

^a^Based on the proportion of horned animals in each genotype class

**Fig. 2:**
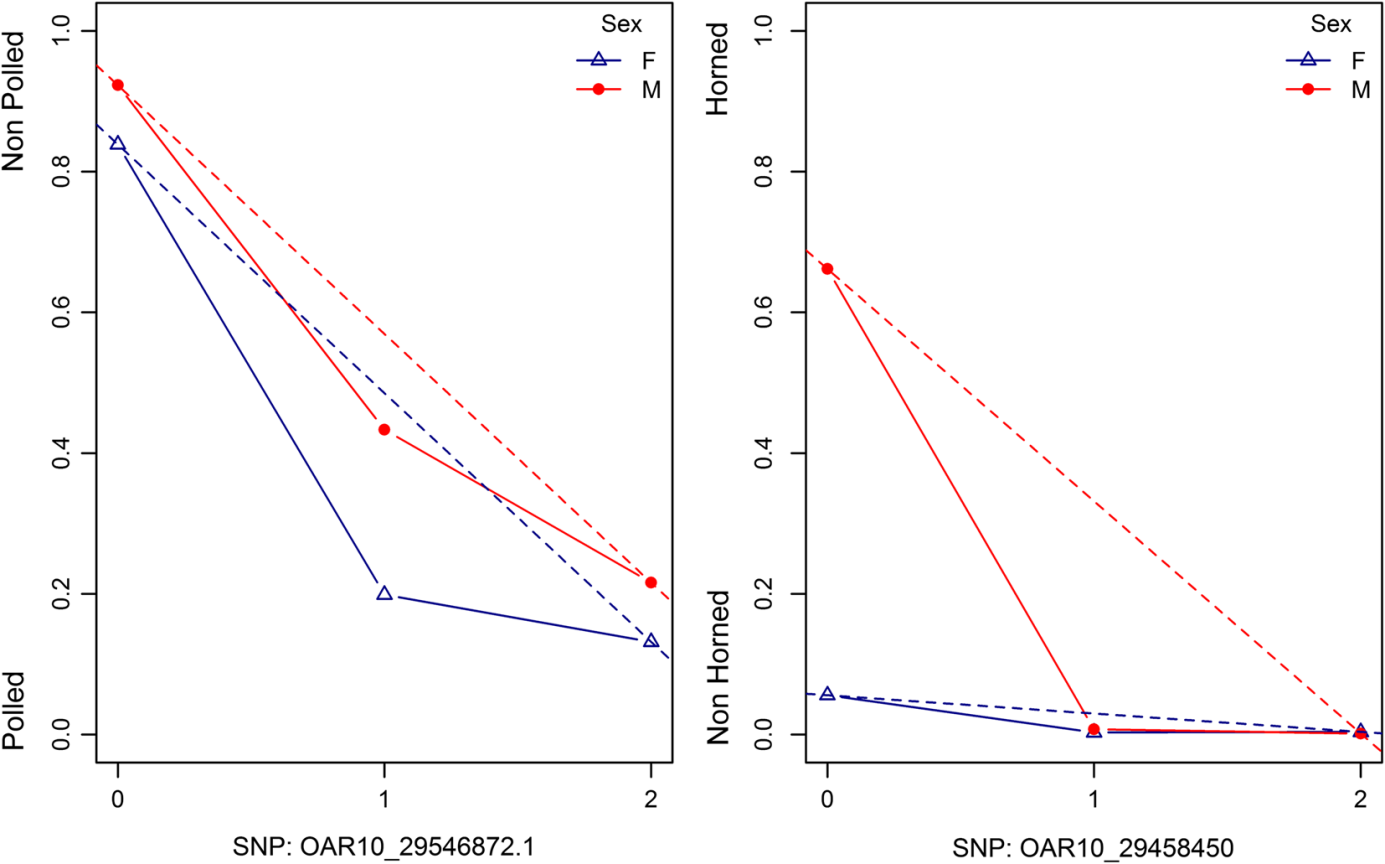
Degree of dominance per sex. **a** Degree of dominance for polled/non-polled. Non-polled is coded as 1 and polled as 0. The dotted line indicates an additive model, where the solid line includes dominance (best fitted model). The lines for females and males are blue and red, respectively. **b** Degree of dominance for horned/non-horned. Horned is coded as 1 and non-horned as 0. The dotted line indicates an additive model, where the solid line includes dominance (best fitted model). The lines and males are blue and red, respectively

Table 4 shows the results for the mode of inheritance for P/NP and H/NH. For P/NP, the best model included an additive and sex-dependent effect for dominance. For H/NH, the best model included sex-dependent additive and dominance effects (i.e. a sex-by-gene interaction). Figure 2 shows the degree of dominance per sex for P/NP and H/NH. H/NH showed complete dominance in males, whereas P/NP showed incomplete dominance in males and females.

**Table 4:** Testing various modes of inheritance for polled/non-polled (P/NP) and horned/non-horned (H/NH) at the single SNP locus based on Akaike’s information criterion (AIC)

Model^a^	P/NP		H/NH	
	AIC	ΔAIC	AIC	ΔAIC
(1) Additive	− 3387	0	− 5070	0
(2) Additive + dominance	− 3580	193	− 5168	99
(3) Sex-dependent additive + dominance	− 3689	302	− 5162	92
(4) Additive + sex-dependent dominance	− 3759	371	− 6871	1802
(5) Sex-dependent additive + sex-dependent dominance	− 3752	365	− 7236	2166

^a^Model numbers refer to the models described in the Methods section, paragraph “Mode of inheritance”

### Prediction

Mean prediction accuracies and standard deviations across the five replicates and fivefold validation are in Table 5 for H/NH and in Table 6 for P/NP. Prediction using single-SNP models without additional family information (via either pedigree or genomic relationships) resulted in accuracies for P/NP equal to 0.547 and 0.642, in males and females, respectively. For H/NH, the accuracy was much lower in females (0.155) and higher in males (0.714). Adding pedigree relationships in the model led to a substantial increase in prediction accuracy for H/NH in females (0.323), whereas for P/NP, the increase was small i.e. 0.582 and 0.671 in males and females, respectively. Replacing the numerator matrix with a GRM increased accuracies mainly for P/NP, i.e. 0.620 in males and 0.713 in females. A similar increase was not observed for H/NH (accuracies of 0.723 and 0.303 in males and females, respectively).

**Table 5:** Prediction accuracies for the horned/non-horned phenotype with standard errors (SE) of the mean

Model^a^	Ped	Average		SE	
		F	M	F	M
Single SNP	–	0.155	0.714	0.006	0.006
Single SNP	${\mathbf{A}}$	0.323	0.721	0.017	0.006
Single SNP	GRM	0.303	0.723	0.015	0.006
Hap3	${\mathbf{A}}$	0.323	0.725	0.016	0.006
Hap5	${\mathbf{A}}$	0.324	0.723	0.016	0.005
Hap10	${\mathbf{A}}$	0.315	0.687	0.016	0.006
Hap3	GRM	0.270	0.630	0.011	0.007
Hap5	GRM	0.275	0.581	0.012	0.012
Hap10	GRM	0.293	0.691	0.014	0.006
GRM OAR10	GRM	0.216	0.597	0.006	0.006
GRM	GRM	0.248	0.534	0.010	0.008
Insertion	${\mathbf{A}}$	0.296	0.573	0.013	0.008
Insertion + SNP	${\mathbf{A}}$	0.325	0.723	0.016	0.005

^a^Models fitting a single SNP alone or with a polygenic effect based on pedigree relationships (${\mathbf{A}}$), or genomic relationships (GRM). Models using three, five, or ten SNP haplotypes are abbreviated with ‘Hap’ followed by the number of SNPs used to form the haplotype. Models using GRM for prediction are abbreviated with ‘GRM’

**Table 6:** Prediction accuracies for the polled/non-polled phenotype with standard errors (SE) of the mean

Method^a^	Ped	Average		SE	
		F	M	F	M
Single SNP	–	0.642	0.574	0.007	0.006
Single SNP	${\mathbf{A}}$	0.671	0.582	0.006	0.006
Single SNP	GRM	0.713	0.620	0.004	0.005
Hap3	${\mathbf{A}}$	0.671	0.583	0.005	0.006
Hap5	${\mathbf{A}}$	0.676	0.582	0.006	0.006
Hap10	${\mathbf{A}}$	0.549	0.444	0.007	0.008
Hap3	GRM	0.714	0.621	0.004	0.005
Hap5	GRM	0.716	0.619	0.004	0.005
Hap10	GRM	0.700	0.604	0.004	0.005
GRM OAR10	GRM	0.640	0.578	0.003	0.005
GRM	GRM	0.624	0.533	0.004	0.006
Insertion	${\mathbf{A}}$	0.571	0.483	0.005	0.006
Insertion + SNP	${\mathbf{A}}$	0.671	0.582	0.006	0.006

^a^Models fitting a single SNP alone or with a polygenic effect based on pedigree relationships (${\mathbf{A}}$), or genomic relationships (GRM). Models using three, five, or ten SNP haplotypes are abbreviated with ‘Hap’ followed by the number of SNPs used to form the haplotype. Models using GRM for prediction are abbreviated with ‘GRM’

The methods using haplotypes from multiple SNPs (three, five or ten SNPs) did not increase prediction accuracies substantially, compared to single-SNP prediction models. For example, prediction accuracies for P/NP in males using three, five or ten SNP-haplotypes and using a GRM were equal to 0.714, 0.716 and 0.700, respectively, compared to 0.713 when using a single-SNP haplotype. Creating a haplotype from more than five SNPs even decreased prediction accuracy (not significant).

Method (3) used the sum of two GRM (matrix ${\mathbf{G}}$ for additive and matrix ${\mathbf{D}}$ for dominance) based on either all SNPs across all chromosomes or SNPs on OAR10 only. The prediction accuracies using SNPs on OAR10 only were similar to those obtained by fitting a single SNP for P/NP. For example, prediction accuracy for P/NP in females was equal to 0.640 when the **G + D** model based on SNPs of OAR10 was used compared to 0.642 when the single SNP model was used. The accuracies obtained with the **G + D** model were lower for H/NH in males compared to those obtained with single-SNP or multiple-SNP haplotypes (0.597 for OAR10 vs. 0.714 using single-SNP haplotype). In general, the **G + D** model based on all chromosomes resulted in lower prediction accuracies than fitting only the SNPs on OAR10.

Method (54) used the imputed insertion for prediction. This insertion was fitted as a single SNP (coded as 0, 1 or 2) and modeled according to the model with the best mode of inheritance [Table 4, Model (4) for P/NP and Model (5) for H/NH)]. This method gave a low prediction accuracy for P/NP, i.e. 0.571 versus 0.671 in females and 0.483 versus 0.584 in males. All models included a polygenic effect based on pedigree relationships. We obtained similar results for H/NH. When using both the 1.78-kb insertion and the single-SNP, prediction accuracies were very similar to using only the best single SNP for P/NP and H/NH.

The results for across- and within-family prediction are in Table 7. The model including a single-SNP gave the highest correlations compared to using BLUP (by fitting pedigree only) both for across- and within-family prediction. No differences between the correlations for across- and within-family prediction were observed with the **A + D** model (excluding the prediction accuracy for horned females). Although prediction accuracies in across- and within-family comparisons were quite similar, standard errors (SE) on the estimated correlations were higher for the across-family predictions. For example, within-family prediction for females for P/NP using pedigree information had a SE of only 0.013 compared to 0.10 for across-family prediction. Fitting a single SNP greatly reduced the SE of the correlation to values of 0.01 and 0.004 for across- and within-family prediction.

**Table 7:** Prediction accuracies (SE^a^) per sex for horned/non-horned (H/NH) and polled/non-polled (P/NP) for across- and within-family structure

	Sex^b^	Across-family		Within-family	
		POL^c^	A + D^d^	POL	A + D
P/NP	F	0.34 (0.10)	0.65 (0.013)	0.49 (0.013)	0.66 (0.004)
	M	0.36 (0.09)	0.60 (0.010)	0.40 (0.007)	0.57 (0.003)
H/NH	F	0.23 (0.13)	0.17 (0.007)	0.33 (0.07)	0.32 (0.005)
	M	0.36 (0.11)	0.72 (0.007)	0.51 (0.06)	0.72 (0.003)

^a^Standard error of the mean over five replicates

^b^F = females, M = males

^c^POL = only a polygenic effect fitted via pedigree

^d^**A **+ **D** = model with a single SNP, fitting additive + dominance effect and a pedigree

## Discussion

The most significant SNPs for P/NP and H/NH, i.e. OAR10_29546872.1: 29512572 and OAR10_29458450: 29458450, respectively, were very close to the 1.78-kb insertion in the *RXFP2* gene (at position 29456047 through 29457881 on OAR10). OAR10_29546872.1 has been used by the Australian Sheep CRC since 2012 as the main predictive SNP for the polled phenotype in Merino sheep (J. van der Werf, personal communication), with OAR10_29389966_X.1 (reported by Dominik et al. [12]) as a second predictive genotype (in case of a failed genotype with the first SNP). This OAR10_29389966_X.1 SNP was also in the top 10 most significant SNPs in the GWAS of our study. The SNP reported by Johnston et al. [7] (OAR10_29448537.1: 29415140) was not included in the top 100 SNPs of our GWAS. SNP OAR10_29448537.1 is located upstream and SNP OAR10_29546872.1 downstream of the 1.78-kb insertion. In our population, SNP OAR10_29448537.1 had a higher MAF (0.46) than the other two SNPs i.e. 0.30 and 0.29 for OAR10_29458450 and OAR10_29546872.1, respectively. The difference in significance between the SNP reported by Johnston et al. [7] and the most significant SNP in our study is also reflected in the squared correlation r^2^ of 0.19 between OAR10_29448537.1 and OAR10_29546872.1. The difference in allele frequency and LD patterns can result in the selection of other SNPs for genomic prediction between studies in different populations.

### Mode of inheritance

In the literature, different modes of inheritance have been discussed for the polled phenotype in sheep. Differences in the effects of horned and polled alleles for males and females were already suggested by Wood in the 1900s [30] and Dolling in the 1960s specifically for Merino sheep [31–33]. Johnston et al. [7] suggested that the mode of inheritance of horns was dominant in males and additive in females, but could not rule out the possibility that other genetic regions may explain the remaining polygenic variation in horn phenotype. Dominik et al. [12] suggested a dominant maternal imprinted effect both in males and females. Recently, Lühken et al. [9] discussed the inheritance of the insertion at the 3′UTR of *RXFP2* in many breeds with a variety of horn status. Inconsistent results with the insertion are observed mainly for sheep breeds with sex-dependent or variable horn status. In wild Bighorn sheep, a significant sex * QTL interaction was observed for two horn traits that are co-located on OAR10 [34]. In Merino sheep, horn status is sex-dependent, and the occurrence of scurs and knobs is observed especially in females.

Our results indicate that the degree of dominance differs between P/NP and H/NH (Fig. 2). Figure 2 shows the predicted phenotype given its genotype class by including dominance or assuming additivity only. H/NH showed complete dominance in males whereas P/NP showed incomplete dominance in males and females. The effect of sex on P/NP is not large (both show incomplete dominance), on the contrary, Merino females hardly have horns which makes prediction for H/NH in females difficult (Table 5 and Fig. 2). Our findings confirm the importance of dominance in both males and females, but do not confirm any maternal imprinted effect (results not shown). This is the first study to show statistical evidence for sex-dependent differences in the additive and dominance effects for horned and polled phenotypes.

### Prediction

In this section, we discuss three different observations from our study. First, we discuss the importance of a model that explicitly includes SNPs with large effects on the trait, then the genetic architecture of the polled and horned phenotype and the influence of genes outside the *RXFP2* region, and finally the importance of identifying the 1.78-kb insertion for an accurate prediction.

The four methods used to predict polled and horned phenotypes were evaluated based on the correlation between phenotype and predicted phenotype from a fivefold validation. The method with the lowest correlation was GBLUP and the method with the highest correlation was a single-SNP model or a model that fits a haplotype with three highly significant SNPs. When the single-SNP was not explicitly fitted such as in the GBLUP model, prediction accuracy decreased (for H/NH in males, and P/NP in males and females), indicating the importance of explicitly fitting highly significant SNPs close to the known causative mutation. Applying the GBLUP method, which shrinks all SNP effects equally, will result in a lower prediction accuracy in the presence of a large QTL. Similar results were described by Lee et al. [35] who analyzed coat colour in mice, which is affected by a number of known causal loci with large effects. Similar results were also observed in dairy cattle for the *DGAT1* gene that has a large effect on milk fat percentage. Bayesian methods, which select fewer relevant SNPs compared to GBLUP and allow these to explain more variation, outperformed GBLUP in prediction accuracy [36, 37]. Also a study in pigs in which a large QTL for number of teats in pigs was explicitly fitted, resulted in increased accuracy of prediction [38].

For the prediction of both horned and polled phenotypes, including a polygenic effect in the model achieved better prediction accuracies compared to using only a single SNP, with little difference between modeling the polygenic covariance structure via pedigree-based relationships versus genomic relationships. The variance explained by models including a polygenic effect was larger than that explained by models without pedigree information. For the horned trait, variance components of the model indicated that 70% of the phenotypic variance was explained by a single SNP, and this figure increased to 78% when pedigree was also included. Heritability estimated for H/NH on the underlying scale (using the numerator relationship matrix) without fitting the significant SNP was equal to 0.40 and dropped to 0.06 when fitting the significant SNP as a fixed effect (i.e. it was excluded from the genetic variance). For the polled trait, 51% of the phenotypic variance was explained by a single SNP and 70% of the phenotypic variance when pedigree was also included. Heritability estimated for P/NP on the underlying scale (using the numerator relationship matrix) without fitting the significant SNP was equal to 0.60 and dropped to 0.13 when fitting the significant SNP as a fixed effect. These results indicate that the single SNP does not explain all the genetic variance. However, prediction of non-horned sheep is 100% accurate in males when selecting for the *GG* genotype of SNP OAR10_29458450, and almost perfect in females (one *GG* genotyped female was horned). Although selection against horns can be successful, other forms of horns will still exist such as knobs and scurs.

The potential influence of other genes outside the *RXFP2* region was further investigated via within- and across-family validation. Polygenic traits such as milk production or growth tend to show a pattern in which across-family prediction is lower than within-family prediction. Assuming that non-genetic effects (e.g. common environment) are correctly taken into account and that other effects due to population structure such as genetic groups are accounted for, within-family prediction uses both linkage and LD information for prediction, whereas across-family prediction can only depend on LD [35]. In our study, differences between across- and within-family predictions were small (Table 7), which clearly shows that the effect of other genes, captured by the polygenic effect, was not strong for the polled and horned phenotypes [35]. Alternatively, to show the potential influence of other genes, the significant SNP (OAR10_29546872.1 or OAR10_29458450) can be fitted as a fixed effect and then the GWAS is run across all remaining SNPs. None of the GWA plots showed clear and consistent significant associations (see Additional file 1: Figure S1). In addition, we detected no other significant regions outside the *RXFP2* region when sheep with knobs and scurs were tested against horned sheep in the GWAS (see Additional file 2: Figure S2), which indicates that the trait is not polygenic and the majority of the variation is determined by the *RXFP2* region.

In this study, imputation of the 1.78-kb insertion within the *RXFP2* gene [8] did not result in better prediction accuracies. The most likely reason for the lower prediction accuracy is that none of the animals in this study were sequenced and, thus the insertion was not detected directly on the animal itself but rather obtained through imputation. The accuracy of imputation could have been a limiting factor for not reaching a better prediction accuracy. Estimated LD between the insertion and the most significant SNP (OAR10_29546872.1) was estimated at 0.46 for the imputed dataset (N = 4001) and 0.49 for the sequenced dataset (N = 72). Even lower LD estimates of 0.194 were found between the insertion and SNP OAR10_29511510.1 (position 29,476,678) for sheep breeds with a variable horn status [9]. Estimates of LD for polled or horned breeds were much higher (0.635). Low LD between SNPs up- and downstream of the 1.78-kb insertion will result in lower imputation accuracy for the insertion. However, even if the insertion is correctly identified, horned and polled phenotypes are expected not to be 100% accurately predicted. Incomplete penetrance, allelic heterogeneity or other (environmental) interactions could cause variability in the horn status within genotype.

### Implications

Our findings suggest that prediction of polled and horned phenotypes based on DNA polymorphisms is not 100% accurate. However, prediction of horn status based on predictive single SNPs provides close to maximum accuracy and can be used succesfully in sheep breeding programs to reduce the frequency of horned phenotypes. Implementation into a breeding program including a cost–benefit analysis is discussed (Granleese T, Clark SA, Duijvesteijn N, Bradley PE and van der Werf JHJ: Strategies and cost–benefit of selecting for a polled sheep nucleus using DNA testing, submitted). An important factor for a successful breeding program is to ensure accurate phenotype recording. Although the trait seems simple, classifying between horns, scurs, knobs or polled is still a challenge especially with the interference due to castration of males. Clear guidelines for such recording are necessary to improve the overall prediction of horned and polled phenotypes. Recently, a new SNP chip (15k) was developed, which includes SNPs present in the 1.78-kb insertion. Currently, the number of phenotypes and genotypes is not sufficient to evaluate the accuracy of prediction based on these SNPs, although the expectation is that these SNPs could increase the prediction accuracy even more.

## Conclusions

The mode of inheritance for polled and horned phenotypes is sex-dependent. Horned vs. non-horned and polled vs. non-polled in males both show an additive and dominance effect, whereas non-horned is dominant. Prediction of horned females is difficult since this phenotype is rare and no genetic model is clearly favourable, while polled vs. non-polled shows a similar mode of inheritance in females and males (additive + dominance, and polled is dominant).

Prediction of polled and horned phenotypes when using a single SNP gives an accuracy of ~ 0.7. Prediction accuracy is not 100% since the causative mutation was not genotyped, and the model of inheritance is not completely known yet, e.g. incomplete penetrance, allelic heterogeneity or other (environmental) interactions can cause variability of prediction within genotypes. Neither the models including more SNPs by forming haplotypes, nor those fitting a GRM and dominance matrix based on all SNPs (whole genome and OAR10 only) resulted in higher prediction accuracies. Addition of pedigree information via a numerator relationship matrix or a GRM to a single SNP model did result in an increased accuracy, but only in a slight one. However, interaction with or effects of genes outside the *RFXP2* region were not detected. Nevertheless, a breeding program aimed at eredicating horns from Merino sheep by selection on genotype *GG* of SNP OAR10_29458450 or genotype *TT* of SNP OAR10_29546872.1 (all non-horned) could be successful.

## Additional files

**Additional file 1: Figure S1.** Genome-wide association study for polled and horned corrected for the most significant SNP (OAR10_29458450 or OAR10_29546872.1). Description: (a) females, horned/non-horned, (b) males horned/non-horned, (c) females polled/non-polled and (d) males polled/non-polled. The x-axis indicates the genomic location of the SNPs and each chromosome is color-coded. The y-axis shows the −log_10_(*p*-value) of the association statistics for each SNP.

**Additional file 2: Figure S2.** Genome-wide association study for horned vs knobs and scurred sheep. Description: (a) GWAS not corrected for the most significant SNP OAR10_29458450 and (b) GWAS corrected for the most significant SNP OAR10_29458450. The x-axis indicates the genomic location of the SNPs and each chromosome is color-coded. The y-axis shows the −log_10_(*p*-value) of the association statistics for each SNP.
