# Supplementary figures and images for: Genomic prediction of the polled and horned phenotypes in Merino sheep
Source: Genet Sel Evol. 2018 May 22;50:28. doi: 10.1186/s12711-018-0398-6 (PMC5964914; doi:10.1186/s12711-018-0398-6)

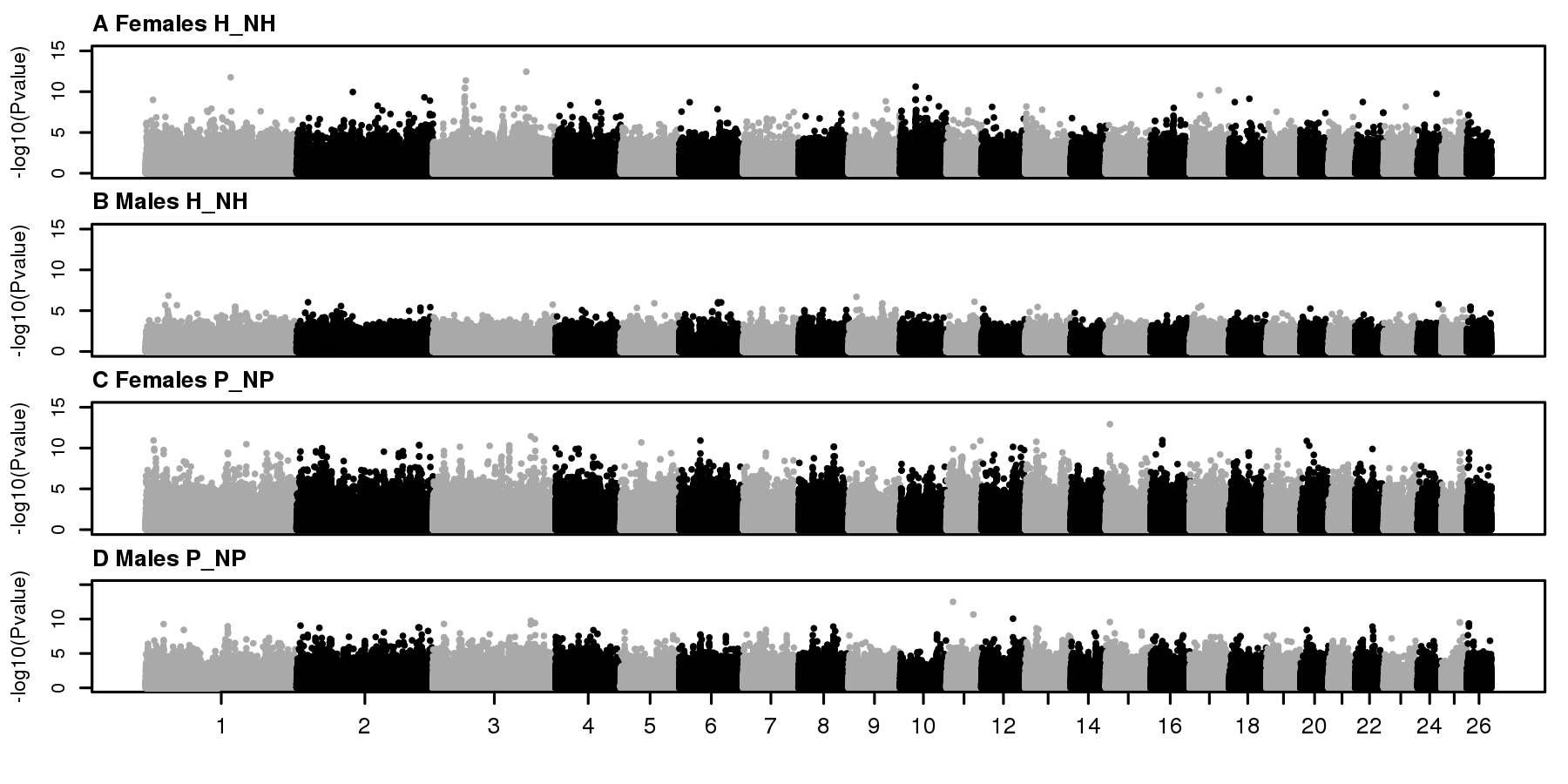

Supplement: Supplementary file 1 — Additional file 1: Figure S1. Genome-wide association study for polled and horned corrected for the most significant SNP (OAR10_29458450 or OAR10_29546872.1). Description: (a) females, horned/non-horned, (b) males horned/non-horned, (c) females polled/non-polled and (d) males polled/non-polled. The x-axis indicates the genomic location of the SNPs and each chromosome is color-coded. The y-axis shows the −log10(p-value) of the association statistics for each SNP. [file 12711_2018_398_MOESM1_ESM.png]

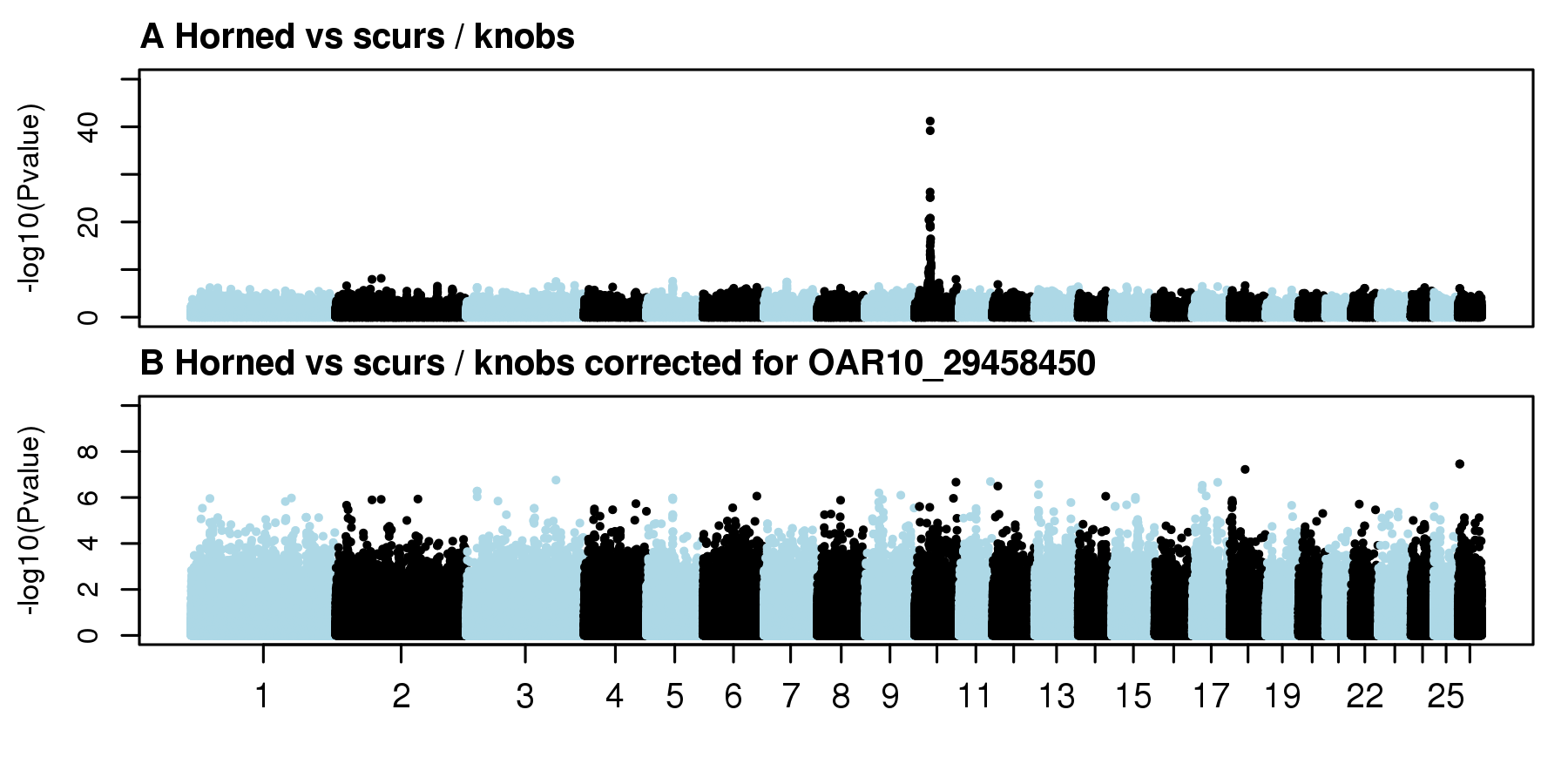

Supplement: Supplementary file 2 — Additional file 2: Figure S2. Genome-wide association study for horned vs knobs and scurred sheep. Description: (a) GWAS not corrected for the most significant SNP OAR10_29458450 and (b) GWAS corrected for the most significant SNP OAR10_29458450. The x-axis indicates the genomic location of the SNPs and each chromosome is color-coded. The y-axis shows the −log10(p-value) of the association statistics for each SNP. [file 12711_2018_398_MOESM2_ESM.png]
